# Development of a novel glycoengineering platform for the rapid production of conjugate vaccines

**DOI:** 10.1186/s12934-023-02125-y

**Published:** 2023-08-18

**Authors:** Sherif Abouelhadid, Elizabeth R. Atkins, Emily J. Kay, Ian J. Passmore, Simon J. North, Burhan Lehri, Paul Hitchen, Eirik Bakke, Mohammed Rahman, Janine T. Bossé, Yanwen Li, Vanessa S. Terra, Paul R. Langford, Anne Dell, Brendan W. Wren, Jon Cuccui

**Affiliations:** 1 Department of Infection Biology, London School of Hygiene & Tropical Medicine, London, WC1E 7HT UK; 2 Department of Life Sciences, Imperial College London, London, SW7 2AZ UK; 3 Department of Infectious Diseases, Imperial College London, London, W2 1NY UK

**Keywords:** Glycoengineering, Bioconjugation, Conjugate vaccines, Bacterial vaccines

## Abstract

Conjugate vaccines produced either by chemical or biologically conjugation have been demonstrated to be safe and efficacious in protection against several deadly bacterial diseases. However, conjugate vaccine assembly and production have several shortcomings which hinders their wider availability. Here, we developed a tool, Mobile-element Assisted Glycoconjugation by Insertion on Chromosome, MAGIC, a novel biotechnological platform that overcomes the limitations of the current conjugate vaccine design method(s). As a model, we focused our design on a leading bioconjugation method using *N-*oligosaccharyltransferase (OTase), PglB. The installation of MAGIC led to at least twofold increase in glycoconjugate yield via MAGIC when compared to conventional *N-*OTase based bioconjugation method(s). Then, we improved MAGIC to (a) allow rapid installation of glycoengineering component(s), (b) omit the usage of antibiotics, (c) reduce the dependence on protein induction agents. Furthermore, we show the modularity of the MAGIC platform in performing glycoengineering in bacterial species that are less genetically tractable than the commonly used *Escherichia coli*. The MAGIC system promises a rapid, robust and versatile method to develop vaccines against serious bacterial pathogens. We anticipate the utility of the MAGIC platform could enhance vaccines production due to its compatibility with virtually any bioconjugation method, thus expanding vaccine biopreparedness toolbox.

**Supplementary Information:**

The online version contains supplementary material available at 10.1186/s12934-023-02125-y.

## Introduction

For more than half a century, antibiotics were considered the first line of defence against bacterial pathogens [1]. However, the spread of antibiotic resistance amongst pathogenic bacteria entails considerable efforts to look for antibiotic alternatives to avoid a foreseeable health crisis. Vaccines have been successful in curbing infectious diseases for decades, not only among adults but also among children and the elderly, thus saving millions of lives worldwide [2].

Glycoconjugate vaccines are considered to be one of the safest and most effective tools to combat serious infectious diseases including bacterial diarrhea, meningitis, and pneumonia [3]. Conjugation is achieved by linking glycans (carbohydrate moiety), either chemically or enzymatically, to proteins via covalent bonds. This leads to a T-cell dependent immune response, offering excellent immune protection in people of all ages [4] Traditionally chemical approaches to produce glycoconjugate vaccine involve the activation of functional groups on the glycan and protein that are linked chemically in a multi-step method that is expensive, laborious, and require several rounds of purification after each step [5]. Additionally, chemical conjugation methods such as reductive amination can alter the polysaccharide epitope, affecting the immunogenicity of the glycoconjugate against the disease, besides its inherent batch-to-batch variation [6].

Biological conjugation (bioconjugation) offers a low-cost alternative to chemical conjugation that is flexible in vaccine design. It is based on using a bacterial cell, usually *Escherichia coli,* as a chassis to express a pathway that encodes the desired bacterial polysaccharide, carrier protein, and an oligosaccharytransferase enzyme, OST, that catalyses conjugation of the carrier protein to polysaccharide [5]. Although, the advent of the bacterial bioconjugation method allowed several protein glycan vaccine combinations to be successfully developed, some of which are currently in clinical trials [7]. However, several challenges hinder the immense potential of bioconjugation to become the preferred method to develop future glycoconjugate vaccines. Firstly, the process places significant metabolic stress on the *E. coli* vaccine micro-factory, due to the expression of orthogonal pathways [8]. This process requires the prior genetic and structural information of the polysaccharide structure of choice. Secondly, the use of three independent replicons has limitations due to the incompatibility of plasmid origins of replication and antibiotic selection markers. This can lead to plasmid loss and result in reduction of glycoconjugate yield [7, 9, 10]. Thirdly, reports have demonstrated that the expression of the OTase PglB, that catalyses the linking of glycans to carrier proteins, has a detrimental effect on bacterial growth, thus decreasing cellular fitness to produce glycoconjugates [8, 11]. Collectively this results in a low biomass which often translates to a reduction in the vaccine yield. Consequently, this leads to an increase in the production cost of a glycoconjugate vaccine, making it unaffordable in low-income countries where they are most needed, putting millions of lives at risk as a result of vaccines inequity [5].

Previous attempts to engineer robust glycoengineering host strains using homologous recombination have had limited success [11]. Although this technology managed to moderately boost glycoprotein production and reduce the dependence on plasmids, it suffers from major drawbacks. Firstly, the method is slow and requires the successful expression of recombinase systems from plasmids to allow chromosomal integration of glycoengineering components. Secondly, it cannot be applied to other Gram-negative bacteria since prior knowledge of the genome sequence is required to allow for the design of homologous arms for homologous recombination to occur. Thirdly, the scarcity of genetic manipulation tools, which are available for limited bacteria, impede the wide use of homologous recombination platforms.

Here, we present a novel platform to overcome some of the limitations of bioconjugation by creating a modular system to rapidly develop conjugate vaccine candidates. This platform could be biologically tailored in a “plug-and-play” manner to allow the integration and stable expression of the glycoengineering component(s) not only in *E. coli* but also in other Gram-negative bacteria. We term this technique, Mobile-element Assisted Glycoconjugation by Insertion on Chromosome (MAGIC). At first instance, we sought to assess the applicability of MAGIC with the commonly used *N-*OTase bioconjugation method. We show how this platform enables the rapid assembly of stable glycoconjugate combinations. We also report that once a bacterial cell has undergone the MAGIC process, it can be used in its own right as a chassis strain to achieve superior glycoconjugate yields and higher glycosylation efficiency when compared to the traditional three plasmid-based bioconjugation methods, and cell free glycosylation method. Furthermore, integration of the OTase into host *E. coli* cells was shown to alleviate much of the metabolic burden from the bacterium that was demonstrated as a direct increase of biomass and glycoconjugate yield. In addition, we present the versatility and robustness of MAGIC beyond glycoengineering *E. coli* strains and in other genetically less tractable host bacteria such as *Citrobacter* species*.* To illustrate the potential application of MAGIC as a rapid method to develop a candidate conjugate vaccine, we speculated a scenario of an *E. coli* O157 outbreak. Then we utilised MAGIC to successfully develop a candidate glycoconjugate vaccine via linking O157 O-antigen to a model carrier protein. The method allowed a glycoconjugate to be created in approximately a week. This also demonstrated that MAGIC could be used in developing candidate glycoconjugates without prior knowledge of bacterial polysaccharides structure and when genetic manipulation tools are scarce. The modular nature of MAGIC highlights its applicability as a tool for biopreparedness especially against emerging multi-drug resistant bacteria.

## Results

### Assessment of MAGIC in developing a conjugate vaccine against *Francisella tularensis*

To demonstrate the proof-of-principle of MAGIC application in glycoconjugate production, we first constructed *E. coli* MAGIC v.1, based on inducible *pglB* under a P_tac_promoter system [12]. PglB is responsible for decorating more than 50 proteins in *Campylobacter jejuni* with a heptasaccharide (GalNAc: -α1,4-GalNAc-α1,4-GalNAc-[Glcβ1,3-]GalNAc-α1,4-GalNAc-α1,4-GalNAc-α1,3-Bac-β1, where GalNAc is *N-*acetylgalactosamine, Glc is glucose, diNAcBac is 2,4-diacetamido-2,4,6-trideoxyglucopyranose) attached to the asparagine residue in the acceptor sequon D/E-X_1_-N-X_2_- S/T with X_1_ and X_2_ as any amino acid except proline. The successful expression of PglB was instrumental linking bacterial polysaccharides to selected carrier proteins in *E. coli.* This led to the development of low-cost vaccines candidates against several deadly diseases. Since it is considered as the most studied and utilised bacterial OTase, therefore it would serve as an exemplar to test MAGIC. We applied MAGIC v.1 to all major glycoengineering strains such as, *E. coli* W3110, *E. coli* SDB1, *E. coli* SΦ874, *E. coli* SCM3, *E. coli* SCM6, *E. coli* SCM7, and *E. coli* CLM24. This was achieved by overnight conjugation, and subsequent culturing, under antibiotic selection, to confirm *pglB* integration on the chromosome. The stability of this integration was confirmed by subculturing *E. coli* MAGIC v.1 more than 10 times without any antibiotic selection and demonstrating that the insertion site had remained intact (data not shown). We then tested *E. coli* MAGIC v.1 in developing a vaccine against *Francisella tularensis* SchuS4. The bacterium *F. tularensis* is categorized as a scheduled bioterrorism class A threat agent due to its high infectivity, low infectious dose, and ease of aerosol distribution [13]. The O-antigen of *F. tularensis*, designated here as Ft O-Ag, consists of the repeating unit of the tetrasaccharide [2)-β-Qui4NFm-(1 → 4)-α-GalNAcAN-(1 → 4)-α-GalNAcAN-(1 → 3)-β-QuiNAc-(1 →]. Previous studies demonstrated that rodents vaccinated by either Ft-LPS or Ft O-Ag glycoconjugate were protected against *F. tularensis* infection [9]. Initially, we established *E. coli* as a positive control, designated here as *E. coli* gCmeA bioconjugation Ft O-Ag, expressing Ft O-Ag biosynthetic pathway, PglB under P_tac_ promoter from pEXT21, and CmeA as a model carrier protein. The periplasmic accessory protein CmeA glycoprotein from *Campylobacter jejuni* has traditionally been used as a model carrier protein to investigate glycoengineering components. The glycoprotein carries two native glycosylation sites, ^121^DFNRS^125^ and ^271^DNNNS^275^ [14]. A C-terminal 6xHis tag was added to CmeA to facilitate its purification via immobilised metal ion chromatography (IMAC) and Western blot analysis. We used a *waaL* deficient *E. coli* strain, CLM24, considered optimal for glycoconjugate production since the O-antigen ligase gene, *waaL,* is deleted, preventing competition with PglB for UndPP linked substrate [15].

**Fig. 1:**
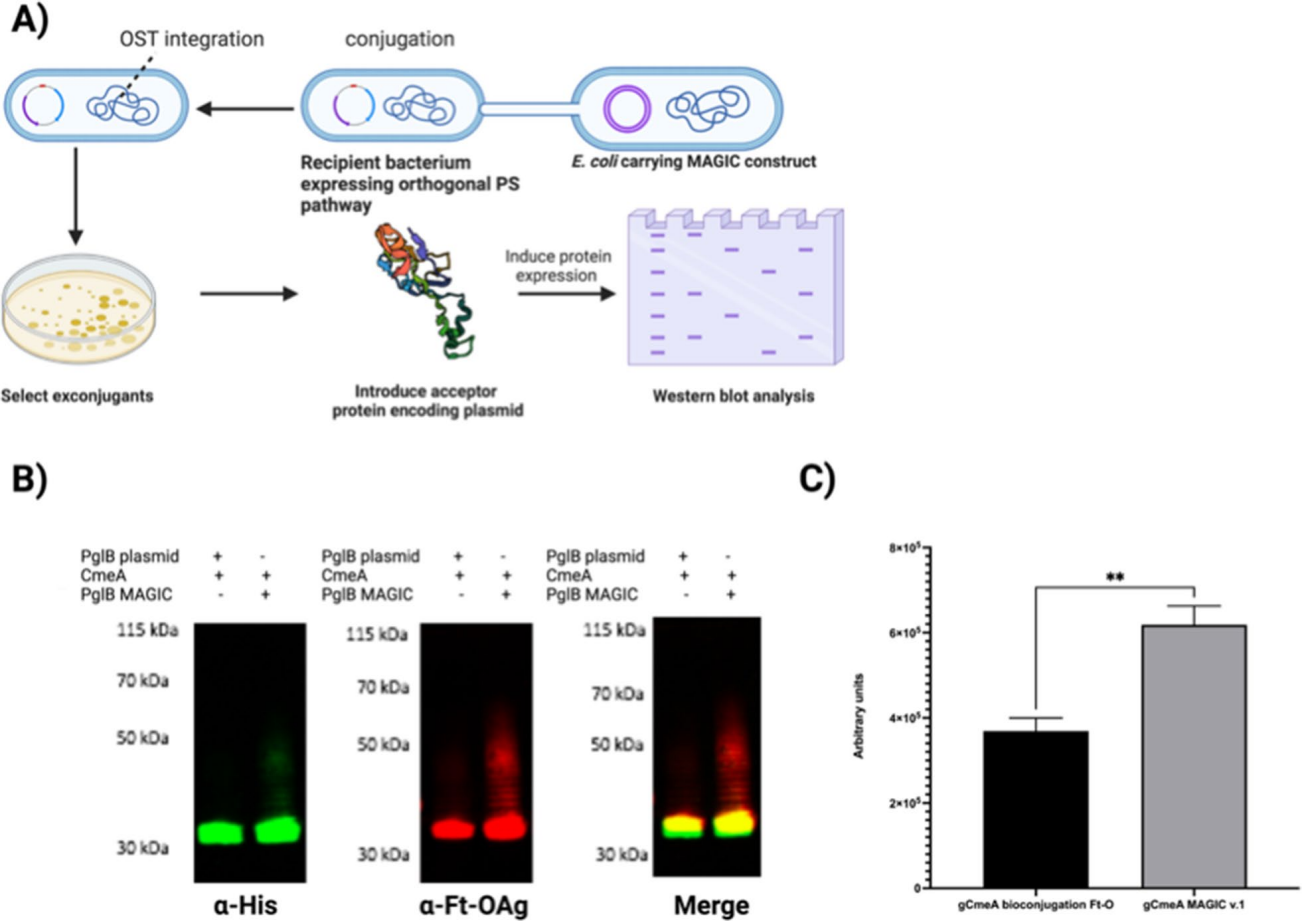
Glycoconjugate production in *E. coli* MAGIC strains compared to conventional bioconjugation method. **A** Schematic diagram of developing of constructing *E. coli* MAGIC; **B** Western blot of 5 µg His-tagged CmeA protein purified by nickel affinity chromatography. Biological samples were separated on a Bolt 4–12% bis–tris gel (Invitrogen) with MOPS buffer and transferred to nitrocellulose membrane with an iBlot 2 dry blotting system. The membrane was probed with anti-His (Invitrogen) and anti- Ft-O antigen monoclonal antibody (Abcam) and detected with fluorescently labelled secondary antisera (green-His, red-Ft-O-antigen) on a LiCor Odyssey scanner.; **C** densitometry analysis of glycoconjugate production in *E. coli* MAGIC v.1 Ft-O compared to *E. coli* bioconjugation Ft-O. Densitometry analysis of glycoconjugate was done from three biological replicates. Statistical analysis is from three biological replicates using Student’s *t-*test ns*, p* > 0.05; *,p < 0.05, **,*p* < 0.01, ***, *p* < 0.001

We assembled *E. coli* CLM24 MAGIC v.1, designated here as gCmeA MAGIC v.1 Ft O-Ag as summarized in Fig. 1A. To assess the performance of *E. coli* CLM24 MAGIC v.1 against gCmeA bioconjugation Ft O-Ag. Both *E. coli* CLM24 variants were grown overnight and subcultured the following day. Bioconjugation was initiated by induction of *pglB* expression at OD_600_ of 0.4–0.5. Under shake flask culture conditions, we demonstrated that *E. coli* gCmeA bioconjugation Ft O-Ag, carrying the three plasmids system, induced with 1 mM IPTG overnight, reached a maximum OD_600nm_ of 1.2 whilst gCmeA MAGIC v.1 Ft O-Ag grew, on average, to an OD_600_ of 1.7 (average of 41% increase in cell density). Western blot analysis of representative CmeA affinity purified from both *E. coli* strains reacted positively when probed with *F. tularensis* O-antigen monoclonal antibody. Interestingly, a visible ladder distinctive of *F. tularensis* O-antigen was observed only in gCmeA MAGIC v.1 Ft O-Ag when probed by either anti-his antibody or O-antigen antibody Fig. 1B. Glycoprotein yield was quantified using image densitometry (glycoprotein/glycoprotein + unglycosylated protein *100) from three biological replicates Additional file 1: Figure S1. An approximate twofold increase in glycoprotein production in gCmeA MAGIC v.1 Ft O-Ag was observed when compared to its counterpart, with glycosylation efficiency increasing from 77.2% ± 14 using traditional three plasmids bioconjugation method to 90.2% ± 2.9 in gCmeA MAGIC v.1 Ft O-Ag Fig. 1C. This degree of polymerization was also not achieved via cell free glycosylation (CFG) method.

**Fig. 2:**
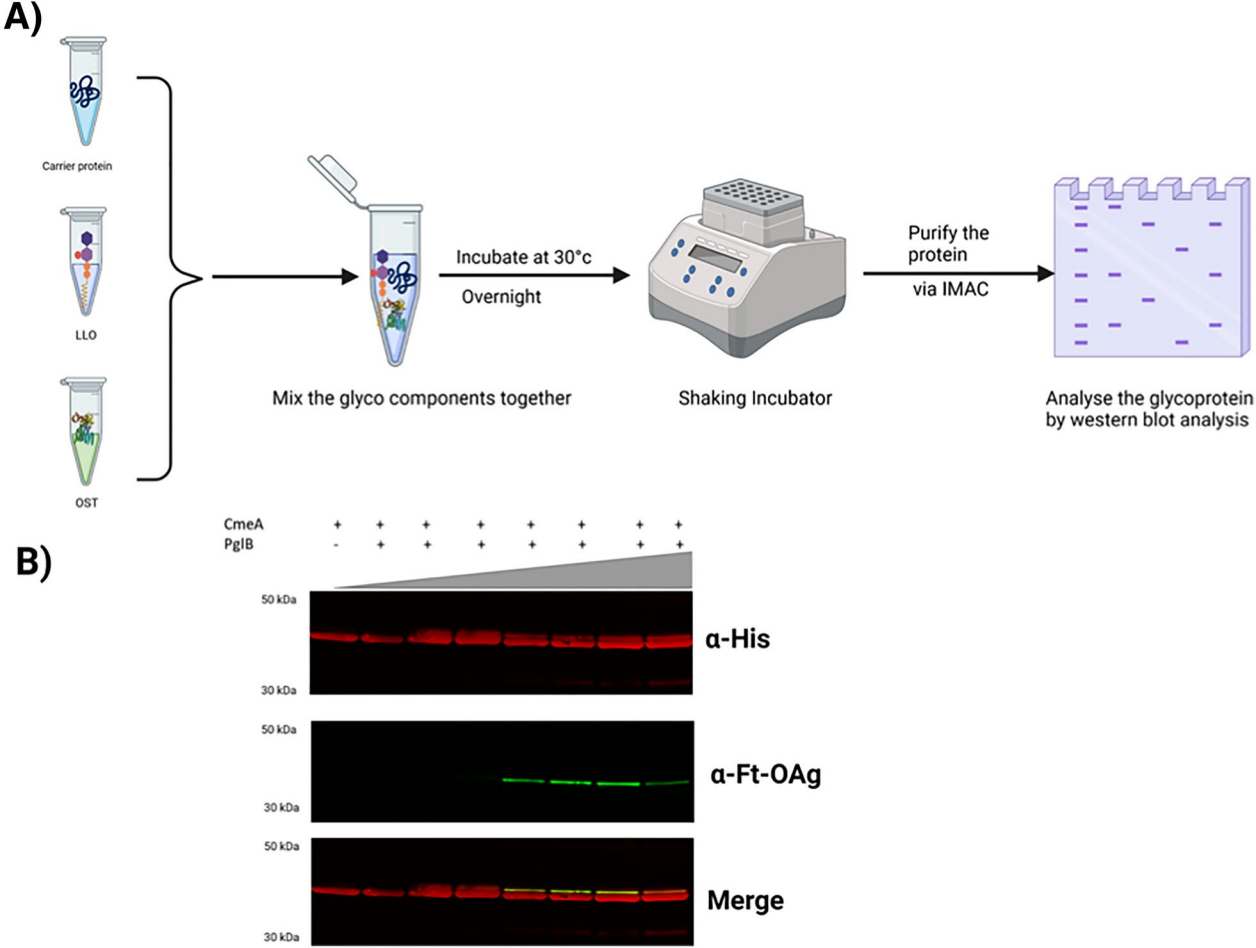
Cell free glycosylation (CFG). **A** schematic diagram of cell free glycosylation analysis; **B** western blot analysis of cell free glycosylation upon gradual increase of cell debris containing glycan donor denoted by the grey triangle. Western blot of 5 µg His-tagged CmeA protein purified by nickel affinity chromatography Protein samples were separated by SDS-PAGE 4–12% bis–tris gel (Invitrogen) with MOPS buffer and transferred to nitrocellulose membrane with an iBlot 2 dry blotting system. The membrane was probed with anti-His (Invitrogen) and anti- Ft-O antigen monoclonal antibody (Abcam) and detected with fluorescently labelled secondary antisera (red-His, green-Ft-O-antigen) on a LI-COR Odyssey scanner

Contrary to performing bioconjugation in bacterial cell, CFG offers a plausible alternative. It is based on expressing main glycoengineering components such as, carrier protein, OTase, and bacterial polysaccharide pathways in *E. coli* cells, then utilise the cell extracts as a source to perform glycosylation in vitro*.* This approach has the flexibility in testing different glycoengineering with various ratios to assess glycosylation efficiency. We performed CFG by expressing each of the glycoengineering component in *E. coli,* cell extracts were obtained by several rounds of cell lysis and enriching for cellular extracts containing CmeA carrier protein and Ft-OAg polysaccharide that were mixed in a constant volume (100 µl). The OTase PglB was then added in an increasing volume of cell extracts (from 20 to 500 µl). The reaction mixture (1 ml) was incubated overnight in shaking incubator at 30 °C followed by carrier protein purification via IMAC. Purified proteins were separated via SDS-PAGE, transblotted, and probed with monoclonal antibody against Ft-OAg and monoclonal antibody against 6xhis Fig. 2A. We observed a faint band that reacted positively with the Ft-OAg antibody upon increasing the volume of cell extracts containing PglB, however, we did not observe any distinct ladder to indicate highly polymerized glycan conjugation to CmeA Fig. 2B (Additional file 2: Figure S2). Additionally, we noticed a reduction in the cell free glycosylation efficiency upon the increase of cell debris containing glycan donor.

Taken together these results show that MAGIC is an effective tool in vaccine production. It also shows that MAGIC can alleviate cellular burdens caused by the glycoengineering component(s) thus allowing for an increased cell density and higher glycoprotein production.

### Improving MAGIC v.1

**Fig. 3:**
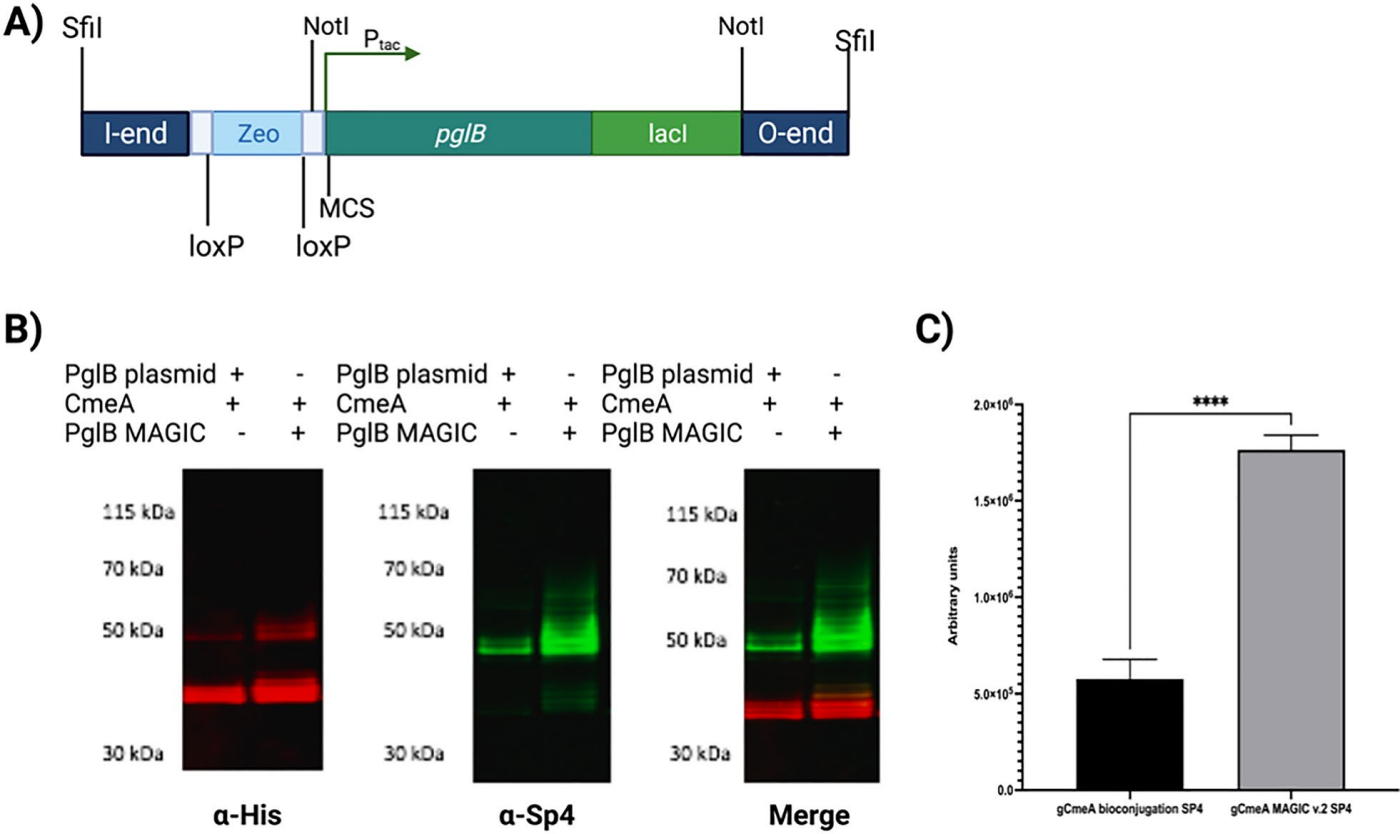
Designing and testing of MAGIC v.2; **A** schematic diagram of I and O end of MAGIC v.2; **B** Western blot of 5 µg His-tagged CmeA protein purified by nickel affinity chromatography. Biological samples were separated on a Bolt 4–12% bis–tris gel (Invitrogen) with MOPS buffer and transferred to nitrocellulose membrane with an iBlot 2 dry blotting system. The membrane was probed with anti-His (Invitrogen) and anti-SP4 (Statens, Serum Institute) and detected with fluorescently labelled secondary antisera (red-His, green- anti-SP4) on a LI-COR Odyssey scanner.; **C** densitometry analysis of glycoconjugate production in *E. coli* MAGIC v.2 SP4 compared to *E. coli* bioconjugation SP4.; Densitometry analysis of glycoconjugate was done from three biological replicates. Statistical analysis is from three biological replicates using Student’s *t-*test ns*, p* > 0.05; *,p < 0.05, **,*p* < 0.01, ***, *p* < 0.001

Guided by these results, we sought to improve MAGIC v.1 by enhancing the assembly process of MAGIC strains. This was achieved by designing a modular system that is more compatible with loading of the glycoengineering component. Secondly, reducing the dependence of antibiotics as a counter selection marker in the glycoconjugate production process. Thirdly, eliminating any unnecessary DNA sequence that increased the size of MAGIC, making chromosomal integration more efficient. To achieve this, we synthesized DNA having the I and O end of MAGIC v.1 and we reduced the cargo size by selecting a small DNA fragment encoding Zeocin^®^ resistance cassette (359 bp), replacing the larger kanamycin antibiotic resistant cassette (816 bp). The Zeocin^®^ resistance cassette was also flanked by *loxp* site to allow removal of antibiotic selection marker once it is integrated on the chromosome. Secondly, we added unique restriction enzyme sites for facile loading of cargo on a high copy pUC vector before transferring onto the chromosomal delivery construct. We designate this as MAGIC v.2 Fig. 3A.

We sought to test MAGIC v.2 in developing a vaccine against the pneumococcal bacterium *Streptococcus pneumoniae*, a leading cause of pneumonia and meningitis worldwide [16] Pneumococcal capsular polysaccharide is one of the main virulence factors and a major component of the vaccine currently in use (PPSV23 and PCV10, 13 and 15). Currently, vaccines against *S. pneumoniae* are produced via chemical conjugation [17]. Previously, we demonstrated that a biologically conjugated vaccine against *S. pneumoniae* confers protection and increased survival rate in laboratory animals [10]. We sought to apply MAGIC v.2 in enhancing the production of a *S. pneumoniae* vaccine. As a control to this experiment, *E. coli* W3110 CmeA-Sp4 was assembled. This strain expresses the orthogonal pathway of *S. pneumoniae* serotype 4 (Sp4), which consists of Pyr-Glc-(1 → 3)-α-ManNAc-(1 → 3)-β-FucNAc-(1 → 3)-α-GlcNAc [18], PglB under P_tac_ promotor from a single copy plasmid pEXT22, and a carrier protein CmeA. Previous attempts to generate a Sp4-CmeA glycoconjugate in CLM24 and W3110 carrying *pglB* expressed from pEXT21 were unsuccessful. We constructed *E. coli* W3110 MAGIC v.2 as detailed in the methods section and removed the antibiotic resistance cassette (Zeocin^®^) using the *cre/loxP* system. Successive subculturing in the absence of antibiotics did not result in the loss of the OST following chromosomal integration with MAGIC v.2. Next, Sp4 and CmeA were added to *E. coli* W3110 MAGIC v.2. Both strains, *E. coli* W3110 Sp4 bioconjugation and *E. coli* W3110 SP4 MAGIC v.2, designated as gCmeA bioconjugation Sp4, and gCmeA MAGIC v.2 Sp4, respectively for ease, were grown overnight in media with shaking, and subcultured the following day. Biological conjugation was initiated by induction of *pglB* expression at OD_600_ of O.4–0.5 by 1 mM IPTG. CmeA 6xHis was purified via IMAC, then analyzed by Western blot analysis. CmeA from Sp4 bioconjugation and MAGIC v.2 Sp4, reacted positively when probed with Sp4 antibody. Western blot analysis showed a distinctive ladder indicative of glycosylation of CmeA by Sp4. Length variability of the glycan polymer was clearly noticed, where gCmeA Sp4 MAGIC v. 2 exhibits a longer polymer when compared to gCmeA bioconjugation Sp4 Fig. 3B and Additional file 3: Figure S3. Image analysis of three biological replicates shows that the CmeA glycoconjugate increased by threefold in MAGIC v.2 when compared to a standard 3 plasmid bioconjugation set-up, with glycosylation efficiency increased from 81.2% ± 7 to 90.4% ± 2.9. This result confirms that glycoprotein production is significantly enhanced by the MAGIC platform Fig. 3C.

**Fig. 4:**
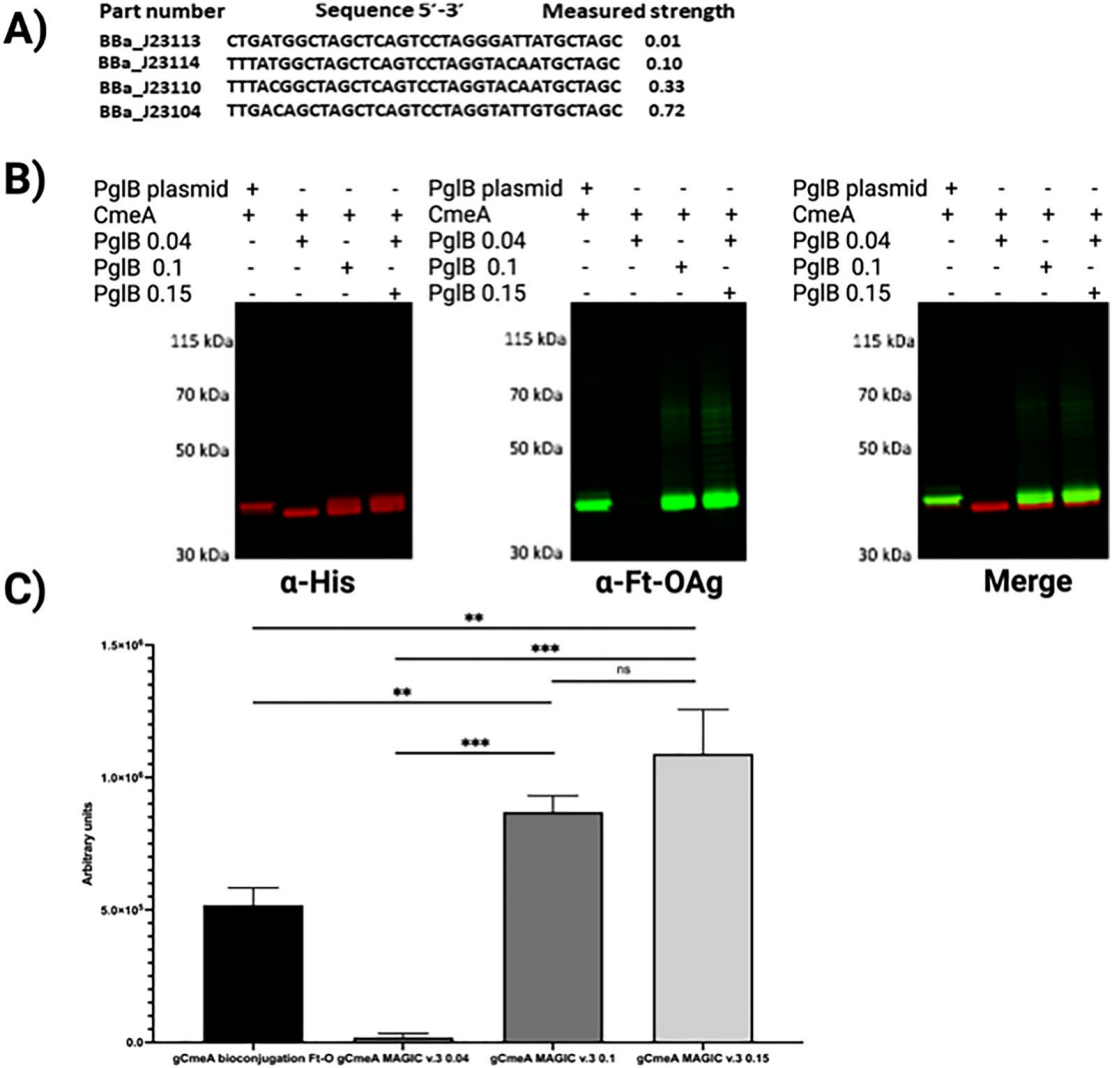
A, Designing and testing MAGIC v.3. **A** nucleotide sequence of Biobricks promoters used in constructing MAGIC v.3 and their corresponding promoter strength [19]; **B **Western blot of 5 µg His-tagged CmeA protein purified by nickel affinity chromatography. Biological samples were separated on a Bolt 4–12% bis–tris gel (Invitrogen) with MOPS buffer and transferred to nitrocellulose membrane with an iBlot 2 dry blotting system. The membrane was probed with anti-His (Invitrogen) and anti- Ft-O antigen monoclonal antibody (Abcam) and detected with fluorescently labelled secondary antisera (green-His, red-Ft-O-antigen) on a LI-COR Odyssey scanner; H, densitometry analysis of glycoconjugate production in *E. coli* MAGIC v.3 Ft-O compared to *E. coli* bioconjugation Ft-O. Densitometry analysis of glycoconjugate was done from three biological replicates. Statistical analysis is from three biological replicates using Student’s *t-*test ns*, p* > 0.05; *,p < 0.05, **,*p* < 0.01, ***, *p* < 0.001

We noticed that the efficiency of *E. coli* MAGIC strains creation was dependent on minimising the size of the cargo within the MAGIC constructs. In order to refine the MAGIC technology, we opted to assemble an OST under control of a constitutive promoter instead of an IPTG inducible promoter. We synthetically designed MAGIC v.3 utilizing σ^70^ promoter(s) variants from Registry of Standard Biological Parts and the iGEM inventory number BBa_J23109 (0.04), BBa_J23114 (0.1), BBa_J23115 (0.15) and BBa_J23104 (0.72), where promoter strength was previously measured in the relative fluorescence units from plasmids expressing RFP in strain TG1 grown in LB media to saturation [19]. Four promoters were chosen to cover a wide range of strength to drive the expression of the OST. Additionally, the inducible promoters assisted in further reducing the distance between the I and O end of the transposon by removing the P_tac_ and *lacI* repressor and potentially further reducing vaccine production cost by omitting the use of a protein induction reagent such as IPTG**.** Next, we assessed the impact of promoter strength on glycoconjugate production yield. We constructed *E. coli* CLM24 MAGIC v.3 variants and tested their relative ability to produce a vaccine against our model O-antigen; *F. tularensis* using CmeA as a carrier protein. Surprisingly, strong promoter BBa_J23104 (0.72) had a deleterious effect on bacterial growth and reduced the glycoconjugate yield when compared to the other three promoters. No growth defects were observed among the other *E. coli* MAGIC v.3 variants (BBa_J23109 (0.04), BBa_J23114 (0.1), BBa_J23115 (0.15) upon overnight culture in liquid media (data not shown). Cells were subcultured the following day and proceeded to IMAC. Western blot analysis showed a higher glycoconjugate yield in CmeA isolated from *E. coli* MAGIC v.3 BBa_J23115 (0.15) (denoted as gCmeA PglB 0.15) when compared to the other promoters and *E. coli* CLM24 CmeA bioconjugation as a control (denoted as gCmeA PglB (0.04), gCmeA PglB (0.1), and gCmeA bioconjugation) Fig. 4A, B. The increase in glycoconjugate yield was reproducible in three biological replicates. Glycoprotein yield was estimated using image densitometry (glycoprotein/glycoprotein + unglycosylated protein *100) from three biological replicates Additional file 4: Figure S4. When compared to the three plasmid bioconjugation method, gCmeA PglB (0.04) showed minimal glycosylation of CmeA. Interestingly, gCmeA PglB (0.1) showed a 1.6-fold increase in glycoprotein yield whilst gCmeA PglB (0.15) showed a twofold increase, when both compared to CmeA bioconjugation Fig. 4C. Glycosylation efficiency of CmeA were 71.9% ± 0.7 in gCmeA bioconjugation method, 74.8% ± 0.9 in gCmeA PglB (0.1), and 74.4% ± 0.6 in gCmeA PglB (0.15) These results demonstrate the importance of fine-tuning glycoengineering components to achieve optimum glycoconjugate yield. Taken together these results demonstrate that MAGIC is a rapid and robust method to advance glycoconjugate production in different glycoengineering *E. coli* strains*.*

### Developing of a candidate conjugate vaccine against *E. coli* O157

One of the most challenging steps in bioconjugation and cell free glycosylation methods is the successful expression of the glycan orthogonal pathway in *E. coli.* This problem is further complicated when ORFs of a certain glycan are scattered on the genome and/or for example when a certain degree of acetylation is necessary for a carbohydrate to be immunogenic [20] and/or the acetyltransferase responsible for this is unknown. In order to overcome this bottleneck, we evaluated the robustness of the MAGIC platform in the development of glycoconjugates in bacteria other than*E. coli*. The bacterium *Citrobacter sedlakii* NRC6070 is a non-pathogenic bacterium that expresses an identical O-antigen to enterohemorrhagic *E. coli* O157, a food-borne pathogen that causes haemolytic-uremic syndrome (HUS) and haemorrhagic colitis, with infectious dose as low as 10^2^ CFU [21, 22]. Antibiotic treatment of *E. coli* O157 could increase the potential risk of development HUS. Phase II clinical trials showed that an O157:H7 O-antigen conjugated to exotoxin A from *Pseudomonas aeruginosa* was safe and immunogenic, as it induced serum IgG LPS antibodies approximately 20-fold higher than pre-vaccination titres after 26 weeks post immunization [21]. The O-antigen consists of the tetrasaccharide repeating unit; α-PerNAc-(1 → 3)- α-Fuc-(1 → 4)- β-Glc-(1 → 3)-α-GlcNAc [22].

**Fig. 5:**
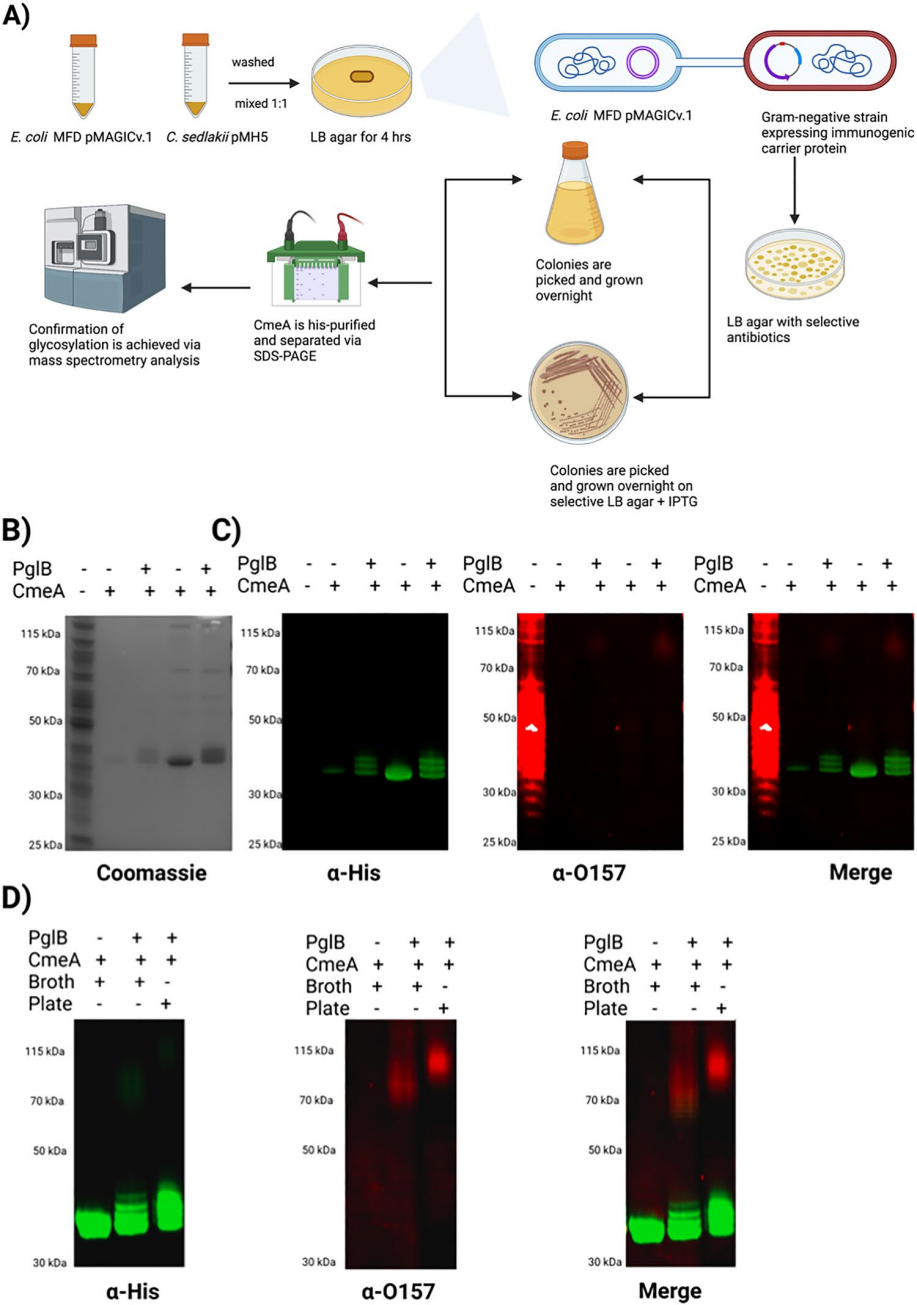
Developing of *E. coli* O157 candidate conjugate in *C. sedlakii* MAGIC v.1. **A** schematic diagram of construction of *C. sedlakii* MAGIC v.1; **B** Coomassie stain of His-tagged CmeA protein purified from *C.sedlakii* and *C. sedlakii* MAGIC v.1 by nickel affinity chromatography. Biological samples were separated on a Bolt 4–12% bis–tris gel (Invitrogen) with MOPS buffer; **C** western blot analysis of CmeA purified from *C. sedlakii* and *C. sedlakii* MAGIC v.1, Biological samples were separated on a Bolt 4–12% bis–tris gel (Invitrogen) with MOPS buffer transferred to nitrocellulose membrane with an iBlot 2 dry blotting system. The membrane was probed with anti-His (Invitrogen) and anti-O157 (Abcam) antibody and detected with fluorescently labelled secondary antisera (green-His, red-O157) on a LI-COR Odyssey scanner. **D** glycosylation of CmeA in *C. sedlakii* MAGIC v.1 in broth and plate of His-tagged CmeA

To assess the versatility and robustness of MAGIC in developing a candidate vaccine in the event of a potential outbreak situation, we aimed at developing a candidate vaccine against foodborne pathogen *E. coli* O157:H7, as an exemplar. We took into consideration two main factors, firstly, to test a rapid method for vaccine development; secondly, to avoid growing a large culture volume of a pathogenic organism which can be a major safety biohazard [23]. Since *E. coli* O157:H7 is categorized as a CAT III organism, we used *C. sedlakii* instead. We developed *C. sedlakii* MAGIC v.1 as described in Fig. 5, A. This time we added a single plasmid expressing our model carrier protein, CmeA 6xHis, as *C. sedlakii* expresses the same O-antigen as *E. coli* O157:H7. The development of *C. sedlakii* MAGIC v.1 expressing CmeA took 3 days in total from streaking the cells in glycerol stocks. To achieve the second aspect, we sought to grow *C. sedlakii* MAGIC v.1 CmeA on 2 LB agar plates supplemented with 5 μM IPTG. The following day, cells were scraped, washed twice with PBS, and CmeA was IMAC purified. Western blot analysis of CmeA isolated from *C. sedlakii* MAGIC v.1 showed an increase in the molecular weight of CmeA in the form of a clear double bands that were not seen in CmeA isolated from *C. sedlakii* wildtype Fig. 5B, C. The increase in mass shift is generally seen as an indication of glycosylation. To confirm this finding, we probed CmeA variants with O157 monoclonal antibody (Abcam), this identified a high molecular weight ladder reacting positively in CmeA isolated from *C. sedlakii* MAGIC v.2 but not in the CmeA *C. sedlaki* wildtype. Interestingly, the double bands did not react with the monoclonal antibody Fig. 5B, C.

**Fig. 6:**
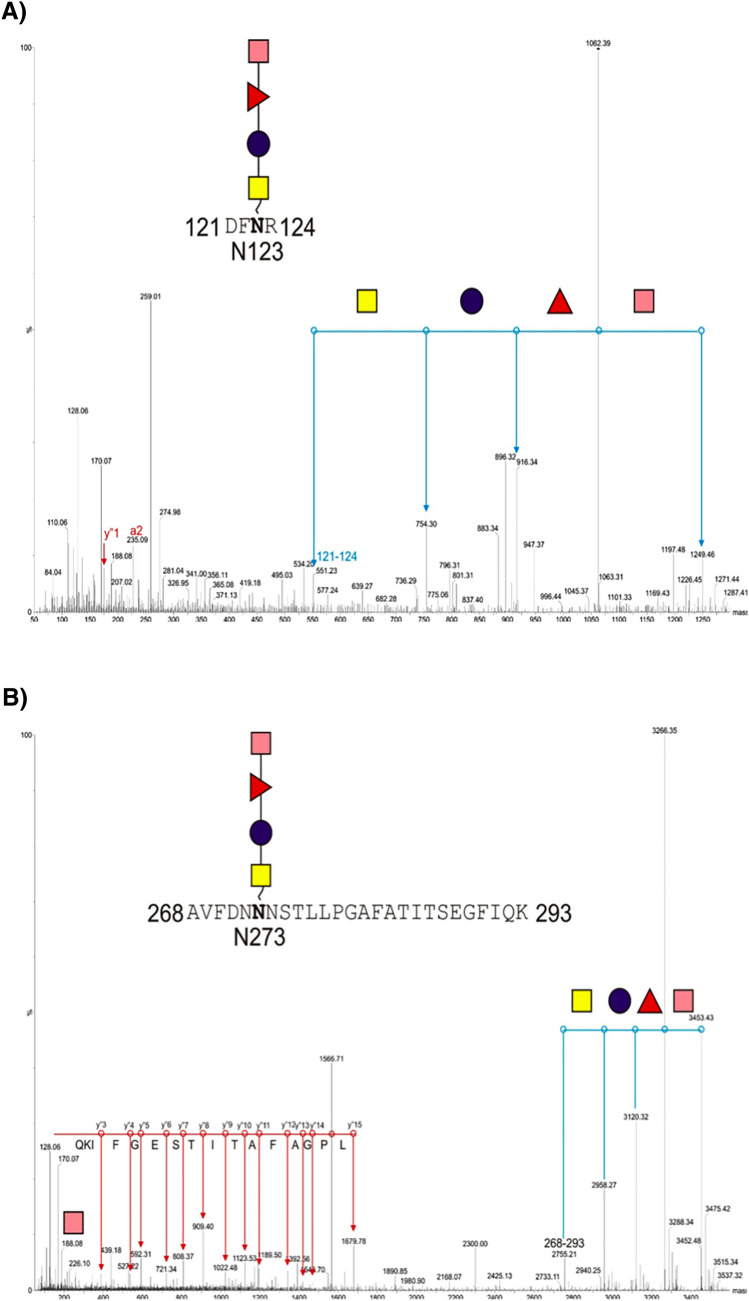
Mass spectrometry analysis of CmeA glycopeptides **A** ^268^AVFDNNNSTLLPGAFATITSEGFIQK^293^; and **B** E^121^DFNR^124^; purified CmeA was reduced, alkenylated, and treated with sequencing grade trypsin overnight, peptides were then run on LC–MS/MS (Waters). Precursor ion fragmentation shows the loss of HexNAc, Hex, deoxyHex, and deoxyHexNAc which corresponds to one repeating unit of O157. Glycan fragmentation is shown in blue lines and peptide fragmentation is shown in red

We used LC–MS/MS analysis to precisely characterize the double bands observed in CmeA *C. seldakii* MAGIC v.2.Gel slices were cut followed by reduction, alkylation, and digested with trypsin. Peptides were separated by LC and detected by CID MS/MS. CmeA was identified after the raw data search. Further data analysis was performed with the addition of O157:H7 repeating unit molecular weight (698 Da) to modification list in the database search method. Two peptides were identified as a part of the D/E-X-N-X-S/T (where X is any amino acid except proline) carrying this glycan ^268^AVFDNNNSTLLPGAFATITSEGFIQK^293^; *m/z* 3452 and ^121^DFNR^124^;*m/z* 1249). Manual analysis of the MS/MS data showed fragmentation of the peptides with characteristic peak (*m/z* 188) in both peptides, which is indicative of PerNAc loss. Further analysis of the peaks identified the modification by a tetramer of 187–146-162–203. The mass of 187 is consistent with an *O-*acetyl deoxyhexose, 146 is consistent with deoxyhexose, 162 is consistent with a hexose, and 203 is consistent with *N-*acetyl hexosamine Fig. 6A, B. These data combined with the Western blot analysis confirm that CmeA is successfully glycosylated with the correct O157 sugar residue by MAGIC v.2.

Next, we sought to assess if there is a difference in glycoconjugate phenotype between an agar plate glycosylation method and the traditional liquid culture media method. Cells were grown, and glycosylation was induced as mentioned in the methods section CmeA 6xHis was affinity purified and analyzed by western blot. CmeA from both culture media and plate showed the distinct extra high molecular weight bands indicative of glycosylation with O157-Ag. Interestingly, in liquid culture media conditions, CmeA exhibited lower polymer length compared to plate glycosylation method. Both of the polymers reacted positively when probed with O157 monoclonal antibody Fig. 5D. These results clearly show that MAGIC is a robust and versatile method that could be used in the development of candidate vaccines against bacterial pathogens in a non-*E. coli* strain. Additionally, we demonstrate that the biotechnology is compatible with health and safety procedures that minimize any biohazard risk.

## Discussion

In this work we designed MAGIC to allow the testing of different bioconjugation components and/or different glycosylation systems, simultaneously, in a “plug-and-play” manner (Additional file 6: Table 1). The utility of MAGIC was assessed by the means of bioconjugation using the commonly used *N-*OTase, PglB. Our results highlight that MAGIC could solve several obstacles in vaccine scalability such as, the choice of plasmids with compatible origin of replications, avoiding antibiotic usage, elimination of induction chemicals, thus reducing the total cost of vaccine production.

Glycan polymer length is one of the factors that could impact vaccines efficacy. In a recent study, large molecular size Ft-OAg polysaccharide glycoconjugate provided superior protection than low molecular size glycoconjugate against intranasal *F. tularensis* challenge in a mouse model of tularemia [24]. Previous attempts to produce highly polymerized glycoconjugates using bioconjugation methods could have been hampered by expressing several glycoengineering components in the cell from plasmids, which consequently, led to lower cellular biomass and glycoconjugate yield. By alleviating the metabolic stress on the cell when applying MAGIC to one or more glycoengineering components, cellular biomass increased by 41% leading to a higher glycoconjugate yield. Densitometry analysis of western blot images of biological triplicate showed less variation in glycoconjugates yield produced via MAGIC strains as well as higher glycan polymerization when compared to traditional bioconjugation method.

In-depth analysis of the vaccine market shows that pneumococcal conjugate vaccine (PCV) is most likely to see a high value growth by 2030, requiring billions of doses to be manufactured [25]. To meet the forecasted market needs, bioconjugation platforms need to be improved to produce higher yields of efficacious glycoconjugate at a lower cost. Bioconjugation is proven to be a powerful tool to develop efficacious vaccines, however, it suffers from the inherent drawback of the necessity of antibiotic usage to maintain replicating plasmids expressing glycoengineering components. Consequently, this could increase glycoconjugate production cost. A key feature of the improved MAGIC v.2 design is the possibility of removing the antibiotic cassette gene without affecting the expression of any of the glycoengineering component(s). This could allow the performance of multiple cycles of MAGIC to the same bacterium using the same antibiotic selection marker*.* A significant threefold increase in glycoconjugate yield with glycosylation efficiency reaching 90.4% ± 2.9 was observed in MAGIC v.2 Sp4 strain compared to CmeA-Sp4 produced via conventional bioconjugation methods. Previously, we demonstrated the efficacy of glycoconjugates produced in MAGIC strains in conferring protection against *S. pneumoniae* when tested in outbred mice [10]*.* Taken together, we set out a novel benchmark in biological conjugation that allows for enhancement of glycoconjugate production with key advantages over the current biological conjugation technologies.

In contrast to the recently published cell free glycosylation method, MAGIC can provide an inexhaustible and renewable source of novel glycoconjugates [26]. This main difference stems from the fact that MAGIC is based on converting the bacterial cell, either *E. coli* or any other Gram-negative bacterium, into a factory for glycoconjugate vaccine production, contrary to the limited reaction volumes in cell free glycosylation [27]. One appealing feature of MAGIC is the reduction of the batch-to-batch variation bottleneck, since no component mixing is required with specific quantities and hence reduce the probability of human error [26]. This in addition to eliminating key steps in cell free methods such as, ultracentrifugation, protein and lipid-linked oligosaccharide quantification [27]. We speculate that the low degree of variation observed when MAGIC is performed is due to the stable expression of the OTase. This could overcome a critical issue such as plasmids segregation as a consequence of expressing three plasmid components.

The need to develop vaccines in an outbreak situation has been dramatically demonstrated on a global scale through the development of vaccines against COVID-19 using mRNA and adenovirus-based technologies. These technology platforms are less suitable for tackling most bacterial infections that have more complex cell surface components. Therefore, we devised a hypothetical bacterial outbreak scenario, and demonstrated that MAGIC could be an indispensable tool in rapidly developing a candidate vaccine. We demonstrated for the first time that glycosylation could be achieved by growing cells on agar plates. This eliminates the need to grow large cultures of pathogens for chemical conjugation and cell free glycosylation. Additionally, it prevents potential health risks associated with culture spillage which could cause a disease outbreak. Our outbreak scenario of developing a candidate vaccine against the major foodborne pathogen *E. coli* O157 was achieved in one week starting from streaking the wildtype strain(s) to purifying a glycoconjugate. This rapid production of glycoconjugate highlights another key feature of the MAGIC platform and its general applicability. Furthermore, this key feature unlocks the potential of bioconjugation beyond *E. coli* strains. Indeed, most of the clinical isolates of pathogenic bacteria are resistant to several antibiotics commonly used as selective marker and in the absence of genetic information of the polysaccharide coding region of a clinical isolate, MAGIC platform could be implemented in an agnostic manner. To test this, we applied MAGIC v.1 to *C. freundii* ballerup 7851/39 expressing CmeA as a carrier protein. Interestingly, we noticed a distinctive polysaccharide ladder cross reacted with the anti-his channel of CmeA isolated from C. freundii MAGIC v.1 Additional file 5: Figure S5. This glycoconjugate was not present in CmeA isolated from C. freundii wildtype and reacted negatively with Vi-CPS monoclonal antibody (Additional file 5: Figure S5).

In summary, we present a novel glycoengineering platform that will accelerate developing a range of glycoconjugate vaccines. The platform provides unique features such as, (a) enhancing bacterial growth rate by decreasing the metabolic burden exerted by glycosylation components on the cell, (b) glycoconjugate yield gains (c) the ability to rapidly generate rationally designed tailor-made vaccines, (d) built-in modularity and compatibility with any glycosylation machinery available, and (e) wide applicability in non-glycoengineering *E. coli* cells*.* Collectively, the application of MAGIC technology could be used in the improvement of existing vaccines, the development of new vaccines, and potentially as a rapid vaccinology response strategy in an infectious disease outbreak situation.

### Supplementary Information

**Additional file 1: Figure S1**. Western blot of 5 µg His-tagged CmeA protein purified by nickel affinity chromatography. Triplicate biological samples were separated on a Bolt 4–12% bis–tris gelwith MOPS buffer and transferred to nitrocellulose membrane with an iBlot 2 dry blotting system. The membrane was probed with anti-Hisand anti- Ft-O antigen monoclonal antibodyand detected with fluorescently labelled secondary antiseraon a LI-COR Odyssey scanner.

**Additional file 2: Figure S2.** Western blot of 5 μg His-tagged CmeA protein purified by nickel affinity chromatography. Triplicate biological samples were separated on a Bolt™ 4-12% bis-tris gel with MOPS buffer and transferred to nitrocellulose membrane with an iBlot 2 dry blotting system. The membrane was probed with anti-His and anti- Ft-O antigen monoclonal antibody and detected with fluorescently labelled secondary anti sera on a LI-COR Odyssey scanner.

**Additional file 3: Figure S3**. Western blot of 5 μg His-tagged CmeA protein purified by nickel affinity chromatography. Triplicate biological samples were separated on a Bolt™ 4-12% bis-tris gel with MOPS buffer and transferred to nitrocellulose membrane with an iBlot 2 dry blotting system. The membrane was probed with anti-His and anti- SP4 antisera and detected with fluorescently labelled secondary anti sera on a LI-COR Odyssey scanner.

**Additional file 4: Figure S4.** Western blot of 5 µg His-tagged CmeA protein purified by nickel affinity chromatography. Triplicate biological samples were separated on a Bolt 4–12% bis–tris gelwith MOPS buffer and transferred to nitrocellulose membrane with an iBlot 2 dry blotting system. The membrane was probed with anti-Hisand/or anti-Vi-CPSand detected with fluorescently labelled secondary antisera on a LI-COR Odyssey scanner.

**Additional file 5: Figure S5.** Western blot of 5 μg His-tagged CmeA protein purified by nickel affinity chromatography. Triplicate biological samples were separated on a Bolt 4-12% bis-tris gel (Invitrogen) with MOPS buffer and transferred to nitrocellulose membrane with an iBlot 2 dry blotting system. The membrane was probed with anti-His (Invitrogen) and/or anti-Vi-CPS (Statens) and detected with fluorescently labelled secondary antisera on a LI-COR Odyssey scanner.

**Additional file 6: Table 1.** Strains and plasmids used in this study.

## Data Availability

All data generated or analysed during this study are included in this published article [and its additional files].

## Abbreviations

Pgl: Protein glycosylation locus
MAGIC: Mobile-element Assisted Glycoconjugation by Insertion on Chromosome
OTase: Oligosaccharyltransferase
COVID: Coronavirus disease
O.D: Optical density
Cme: *Campylobacter* Multidrug efflux
IPTG: (Isopropyl ß-D-1-thiogalactopyranoside)
Glc: Glucose
GlcNAc: N-acetylglucosamine
GalNAc: N-acetylgalactosamine
DiNAcBac: N,N’-Diacetylbacillosamine
GalNAcAN: N-acetylgalacturonic acid
PerNAc: N-acetylperosamine
Qui4NFm: 4,6-Dideoxy-4-formamido-glucose
QuiNAc: N-acetylquinovosamine
FucNAc: N-acetylfucosamine
ManNAc: N-acetylmannosamine

## References

[1] Rosini R, Nicchi S, Pizza M, Rappuoli R (2020). Vaccines against antimicrobial resistance. Front Immunol.

[2] Rappuoli R (2018). Glycoconjugate vaccines: Principles and mechanisms. Sci Transl Med.

[3] Dow JM, Mauri M, Scott TA, Wren BW (2020). Improving protein glycan coupling technology (PGCT) for glycoconjugate vaccine production. Expert Rev Vaccines.

[4] Avci FY, Li X, Tsuji M, Kasper DL (2011). A mechanism for glycoconjugate vaccine activation of the adaptive immune system and its implications for vaccine design. Nat Med.

[5] Kay E, Cuccui J, Wren BW (2019). Recent advances in the production of recombinant glycoconjugate vaccines. npj Vaccines.

[6] Poolman J, Frasch C, Nurkka A, Käyhty H, Biemans R, Schuerman L (2010). Impact of the conjugation method on the immunogenicity of Streptococcus pneumoniae serotype 19F polysaccharide in conjugate vaccines. Clin Vaccine Immunol.

[7] Ihssen J, Kowarik M, Dilettoso S, Tanner C, Wacker M, Thöny-Meyer L (2010). Production of glycoprotein vaccines in Escherichia coli. Microb Cell Fact.

[8] Koffas MAG, Linhardt RJ, Natarajan A, Jaroentomeechai T, Li M, Glasscock CJ (2018). Metabolic engineering of glycoprotein biosynthesis in bacteria. Emerg Top Life Sci.

[9] Cuccui J, Thomas RM, Moule MG, D’Elia RV, Laws TR, Mills DC (2013). Exploitation of bacterial N-linked glycosylation to develop a novel recombinant glycoconjugate vaccine against Francisella tularensis. Open Biol.

[10] Herbert JA, Kay EJ, Faustini SE, Richter A, Abouelhadid S, Cuccui J (2018). Production and efficacy of a low-cost recombinant pneumococcal protein polysaccharide conjugate vaccine. Vaccine..

[11] Strutton B, Jaffé SRP, Pandhal J, Wright PC (2018). Producing a glycosylating Escherichia coli cell factory: the placement of the bacterial oligosaccharyl transferase pglB onto the genome. Biochem Biophys Res Commun..

[12] Dykxhoorn DM, St. Pierre R, Linn T (1996). A set of compatible tac promoter expression vectors. Gene.

[13] Oyston PCF, Sjostedt A, Titball RW (2004). Tularaemia: Bioterrorism defence renews interest in *Francisella*
*tularensis*. Nat Rev Microbiol.

[14] Scott NE, Parker BL, Connolly AM, Paulech J, Edwards AVG, Crossett B (2010). Simultaneous glycan-peptide characterization using hydrophilic interaction 2 chromatography and parallel fragmentation by CID, HCD and ETD-MS applied to the N- 3 linked glycoproteome of *Campylobacter jejuni*. Am Soc Biochem Mol Biol.

[15] Feldman MF, Wacker M, Hernandez M, Hitchen PG, Marolda CL, Kowarik M (2005). Engineering N-linked protein glycosylation with diverse O antigen lipopolysaccharide structures in Escherichia coli. Proc Natl Acad Sci.

[16] Weiser JN, Ferreira DM, Paton JC (2018). *Streptococcus pneumoniae*: transmission, colonization and invasion. Nat Rev Microbiol.

[17] Gao F, Lockyer K, Burkin K, Crane DT, Bolgiano B (2014). A physico-chemical assessment of the thermal stability of pneumococcal conjugate vaccine components. Hum Vaccines Immunother.

[18] Kay EJ, Yates LE, Terra VS, Cuccui J, Wren BW (2016). Recombinant expression of *Streptococcus pneumoniae* capsular polysaccharides in Escherichia coli. Open Biol.

[19] Kelly JR, Rubin AJ, Davis JH, Ajo-Franklin CM, Cumbers J, Czar MJ (2009). Measuring the activity of BioBrick promoters using an in vivo reference standard. J Biol Eng..

[20] Berti F, De Ricco R, Rappuoli R (2018). Role of o-acetylation in the immunogenicity of bacterial polysaccharide vaccines. Molecules.

[21] Rangel JM, Sparling PH, Crowe C, Griffin PM, Swerdlow DL (2005). Epidemiology of Escherichia coli O157:H7 outbreaks, United States, 1982–2002. Emerg Infect Dis..

[22] Vinogradov E, Conlan JW, Perry MB (2000). Serological cross-reaction between the lipopolysaccharide O-polysaccharaide antigens of Escherichia coli O157:H7 and strains of *Citrobcter*
*freundii* and *Citrobacter*
*sedlakii*. FEMS Microbiol Lett..

[23] Bandyopadhyay AS, Singh H, Fournier-Caruana J, Modlin JF, Wenger J, Partridge J (2019). Facility-associated release of polioviruses into communities—risks for the posteradication. Emerg Infect Dis..

[24] Stefanetti G, Okan N, Fink A, Gardner E, Kasper DL (2019). Glycoconjugate vaccine using a genetically modified O antigen induces protective antibodies to *Francisella*
*tularensis*. Proc Natl Acad Sci.

[25] 2020 WHO Global Vaccine Market Report. https://www.who.int/publications/m/item/2020-who-global-vaccine-market-report. Accessed 28 Sep 2021.

[26] Stark JC, Jaroentomeechai T, Moeller TD, Hershewe JM, Warfel KF, Moricz BS (2021). On-demand biomanufacturing of protective conjugate vaccines. Sci Adv.

[27] Schoborg JA, Hershewe JM, Stark JC, Kightlinger W, Kath JE, Jaroentomeechai T (2018). A cell-free platform for rapid synthesis and testing of active oligosaccharyltransferases. Biotechnol Bioeng.

[28] Neuhard J, Thomassen E (1976). Altered deoxyribonucleotide pools in P2 eductants of Escherichia coli K 12 due to deletion of the dcd gene. J Bacteriol..

[29] Alaimo C, Catrein I, Morf L, Marolda CL, Callewaert N, Valvano MA (2006). Two distinct but interchangeable mechanisms for flipping of lipid-linked oligosaccharides. EMBO J.

[30] Garcia-Quintanilla F, Iwashkiw JA, Price NL, Stratilo C, Feldman MF (2014). Production of a recombinant vaccine candidate against *Burkholderia*
*pseudomallei* exploiting the bacterial N-glycosylation machinery. Front Microbiol..

[31] De Lorenzo V, Herrero M, Jakubzik U, Timmis KN (1990). Mini-Tn5 transposoon derivatives for insertion mutagenesis, promoter probing, and chromosomal insertion of cloned DNA in gram-negative eubacteria. J Bacteriol.

[32] Feldman MF, Wacker M, Hernandez M, Hitchen PG, Marolda CL, Kowarik M (2005). Engineering N-linked protein glycosylation with diverse O antigen lipopolysaccharide structures in Escherichia coli. Proc Natl Acad Sci U S A.

