# Supplementary figures and images for: Development of a novel glycoengineering platform for the rapid production of conjugate vaccines
Source: Microb Cell Fact. 2023 Aug 18;22:159. doi: 10.1186/s12934-023-02125-y (PMC10436394; doi:10.1186/s12934-023-02125-y)

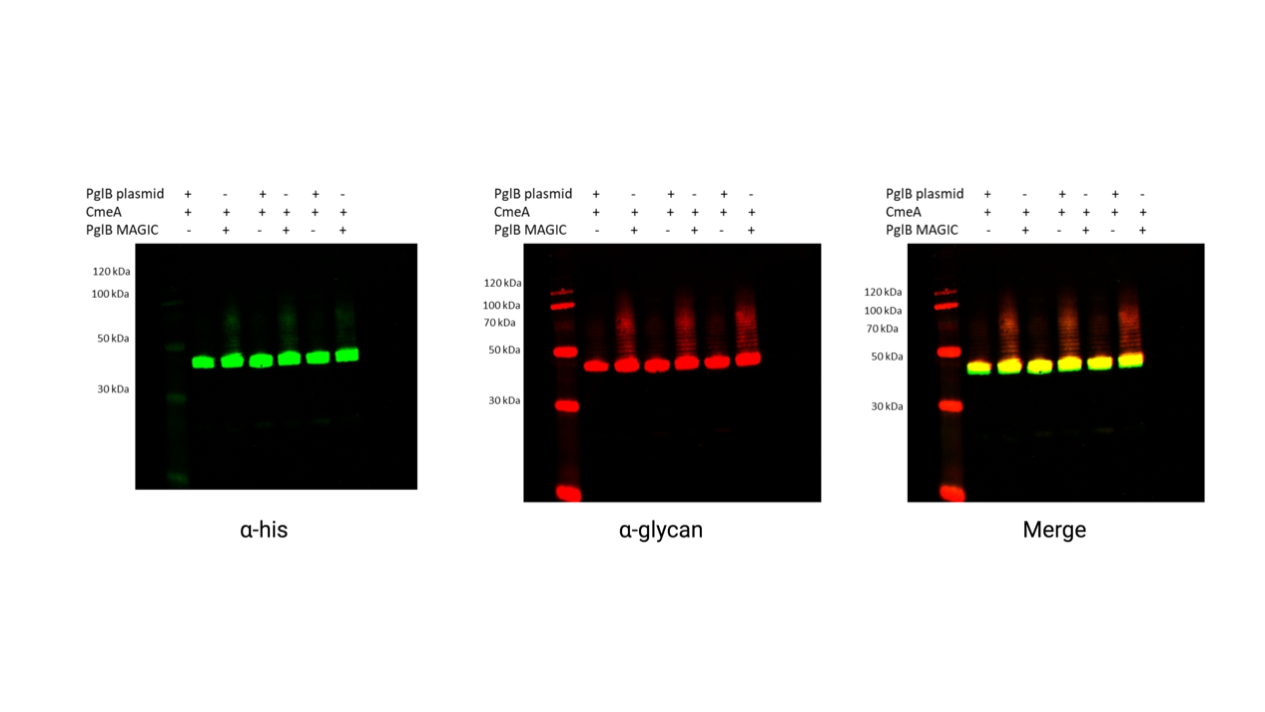

Supplement: Supplementary file 1 — Additional file 1: Figure S1. Western blot of 5 µg His-tagged CmeA protein purified by nickel affinity chromatography. Triplicate biological samples were separated on a Bolt 4–12% bis–tris gelwith MOPS buffer and transferred to nitrocellulose membrane with an iBlot 2 dry blotting system. The membrane was probed with anti-Hisand anti- Ft-O antigen monoclonal antibodyand detected with fluorescently labelled secondary antiseraon a LI-COR Odyssey scanner. [file 12934_2023_2125_MOESM1_ESM.tiff]

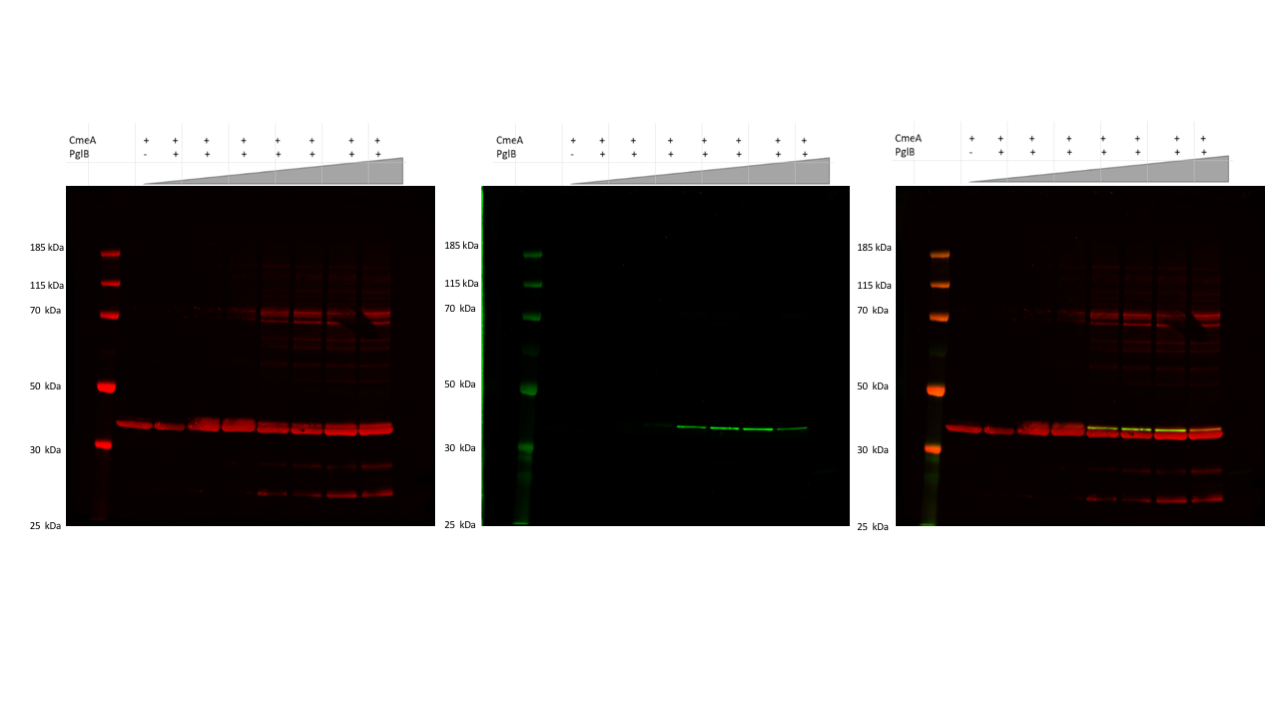

Supplement: Supplementary file 2 — Additional file 2: Figure S2. Western blot of 5 μg His-tagged CmeA protein purified by nickel affinity chromatography. Triplicate biological samples were separated on a Bolt™ 4-12% bis-tris gel with MOPS buffer and transferred to nitrocellulose membrane with an iBlot 2 dry blotting system. The membrane was probed with anti-His and anti- Ft-O antigen monoclonal antibody and detected with fluorescently labelled secondary anti sera on a LI-COR Odyssey scanner. [file 12934_2023_2125_MOESM2_ESM.tiff]

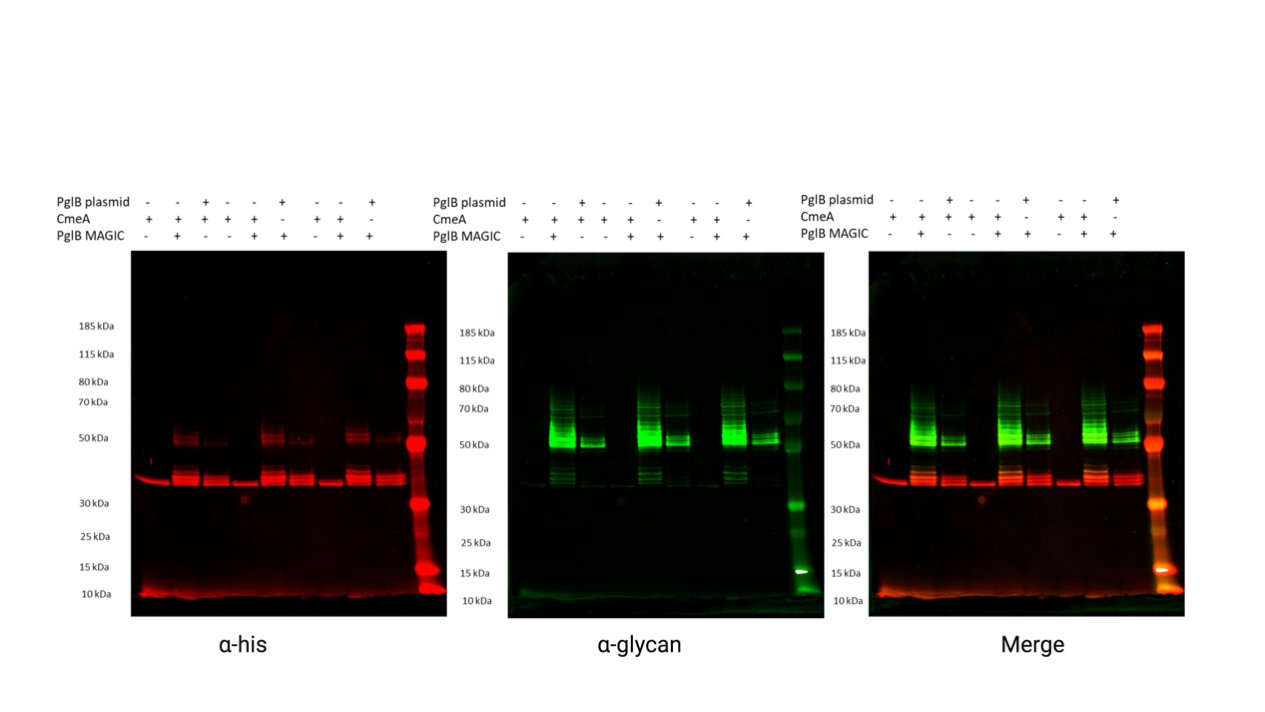

Supplement: Supplementary file 3 — Additional file 3: Figure S3. Western blot of 5 μg His-tagged CmeA protein purified by nickel affinity chromatography. Triplicate biological samples were separated on a Bolt™ 4-12% bis-tris gel with MOPS buffer and transferred to nitrocellulose membrane with an iBlot 2 dry blotting system. The membrane was probed with anti-His and anti- SP4 antisera and detected with fluorescently labelled secondary anti sera on a LI-COR Odyssey scanner. [file 12934_2023_2125_MOESM3_ESM.tiff]

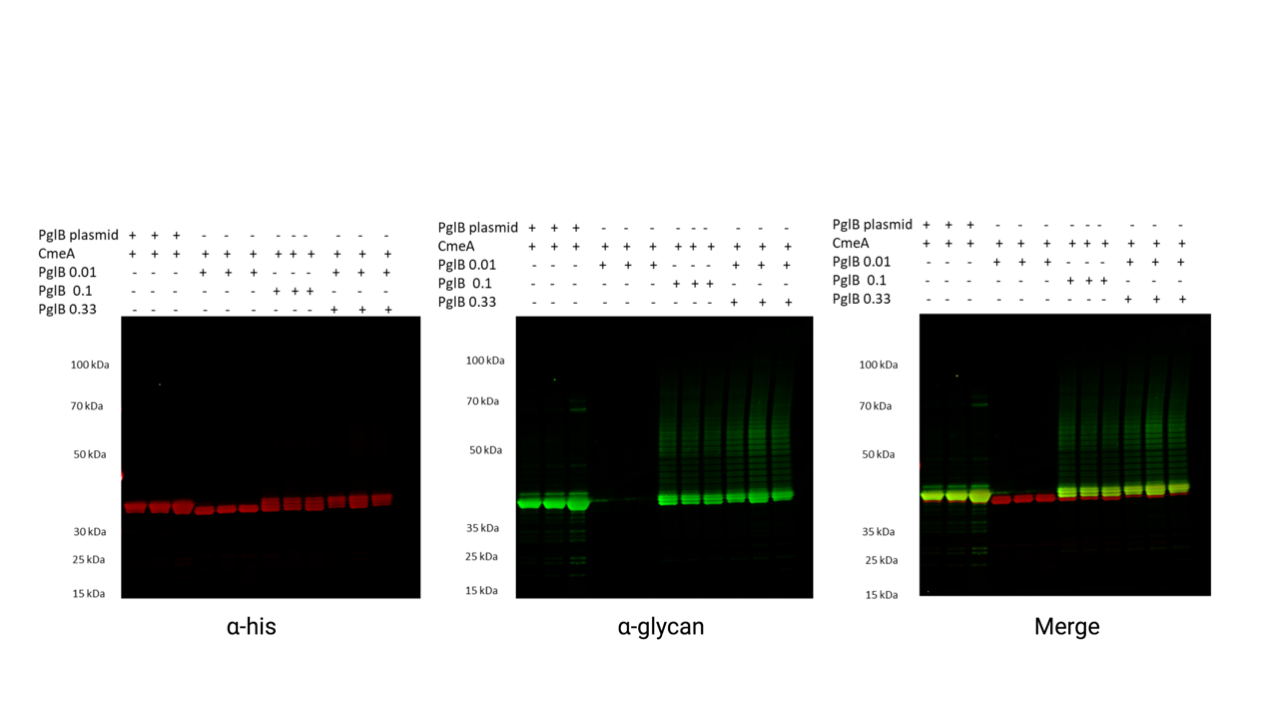

Supplement: Supplementary file 4 — Additional file 4: Figure S4. Western blot of 5 µg His-tagged CmeA protein purified by nickel affinity chromatography. Triplicate biological samples were separated on a Bolt 4–12% bis–tris gelwith MOPS buffer and transferred to nitrocellulose membrane with an iBlot 2 dry blotting system. The membrane was probed with anti-Hisand/or anti-Vi-CPSand detected with fluorescently labelled secondary antisera on a LI-COR Odyssey scanner. [file 12934_2023_2125_MOESM4_ESM.tiff]

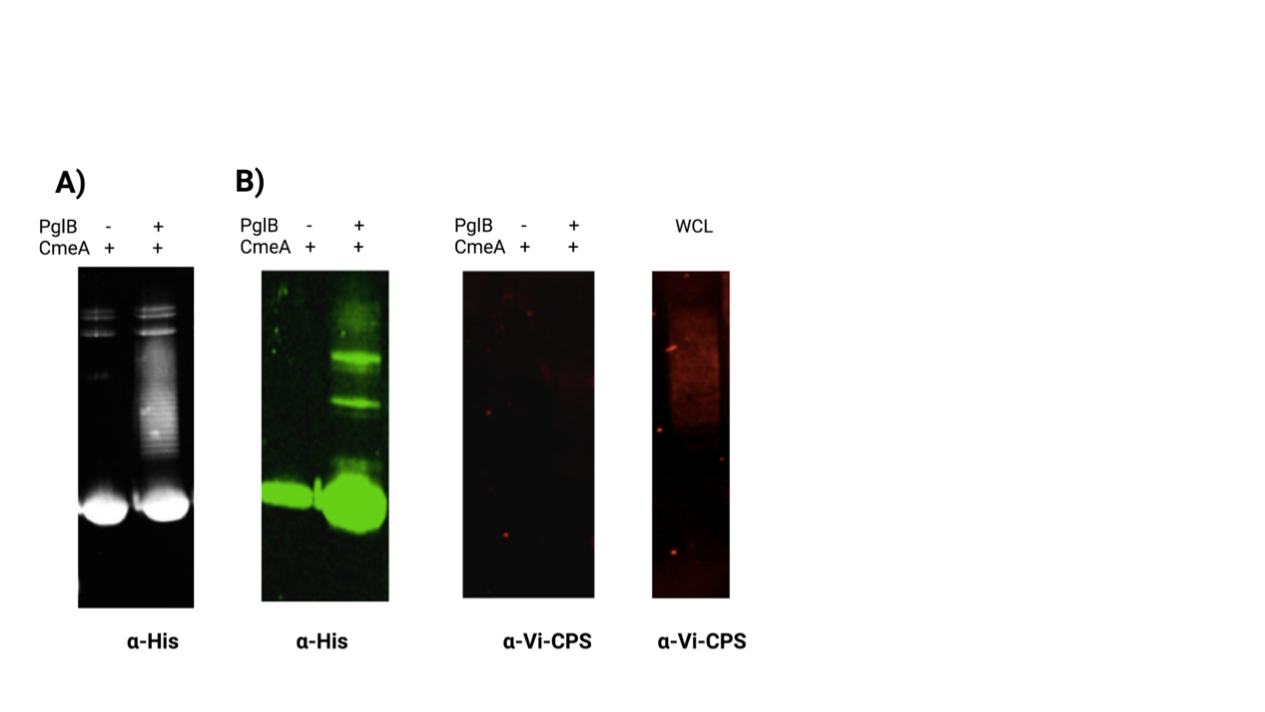

Supplement: Supplementary file 5 — Additional file 5: Figure S5. Western blot of 5 μg His-tagged CmeA protein purified by nickel affinity chromatography. Triplicate biological samples were separated on a Bolt 4-12% bis-tris gel (Invitrogen) with MOPS buffer and transferred to nitrocellulose membrane with an iBlot 2 dry blotting system. The membrane was probed with anti-His (Invitrogen) and/or anti-Vi-CPS (Statens) and detected with fluorescently labelled secondary antisera on a LI-COR Odyssey scanner. [file 12934_2023_2125_MOESM5_ESM.tiff]
