# Supplementary material for: Development of a novel glycoengineering platform for the rapid production of conjugate vaccines
Source: Microb Cell Fact. 2023 Aug 18;22:159. doi: 10.1186/s12934-023-02125-y (PMC10436394; doi:10.1186/s12934-023-02125-y)
Supplement: Supplementary file 6 — Additional file 6: Table 1. Strains and plasmids used in this study. [file 12934_2023_2125_MOESM6_ESM.docx]

**METHODS**

*E. coli* strains were grown on either Luria-Bertani (LB) broth or LB agar, and antibiotics were added when necessary. Bacterial strains used in this study are listed in Table 1

**Table 1**

Strains and plasmids used in this study

| **Strains/Plasmids** | **Description** | **Reference or source** |
| --- | --- | --- |
| *E. coli* DH5α | F- φ80*lac*ZΔM15 Δ(*lac*ZYA-*arg*F) U169 *deo*R*rec*A1 *end*A1 *hsd*R17 (rk-, mk+) *gal-pho*A*sup*E44 λ-*thi*-1 *gyr*A96 *rel*A1 | Thermoscientific (UK) |
| *E. coli* W3110 | *rph-l*IN(*rrnD-rrnE*) | (19) |
| *E. coli* CLM24 | *rph-l*IN(*rrnD-rrnE*) 1Δ*waaL* | (19) |
| *E. coli* SΦ874 | Δ(*sbc-rfb*)*86* O75^-^ | (31) |
| *E. coli* SCM3 | SΦ874 Δ*waaL* | (32) |
| *E. coli* SCM6 | SΦ874 Δ*waaL* Δ*wecA* | Miguel Valvano (Queen’s University, Belfast, UK) |
| *E. coli* SCM7 | SΦ874 Δ*wecA* | Miguel Valvano (Queen’s University, Belfast, UK) |
| *E. coli* SDB1 |  | (12) |
| *Citrobacter sedlakii* | prototroph | ATCC |
| *E. coli* CROW | *rph-l*IN(*rrnD-rrnE*) 1 Δ*waaL ,* Δ*lpxM* | Kay, E *et al* submitted |
| *E. coli* W3110 MAGIC v.1 | *E. coli* W3110 P_tac_­*pglB* kan^r^ | This study |
| *E. coli* CLM24 MAGIC v.1 | *E. coli* CLM24 P_tac_­*pglB* kan^r^ | This study |
| *E. coli* SΦ874 MAGIC v.1 | *E. coli* SΦ874 P_tac_­*pglB* kan^r^ | This study |
| *E. coli* SCM3 MAGIC v.1 | *E. coli* SCM3 P_tac_­*pglB* kan^r^ | This study |
| *E. coli* SCM6 MAGIC v.1 | *E. coli* SCM6 P_tac_­*pglB* kan^r^ | This study |
| *E. coli* SCM7 MAGIC v.1 | *E. coli* SCM7 P_tac_­*pglB* kan^r^ | This study |
| *E. coli* SDB1 MAGIC v.1 | *E. coli* SDB1 P_tac_­*pglB* kan^r^ | This study |
| *Citrobacter sedlakii* MAGIC v.1 | *C. sedlakii* P_tac_­*pglB* kan^r^ | This study |
| *E. coli* CROW MAGIC v.3 | (*pglB* is controlled by BBa_ BBa_J23109  , BBa_J23114, BBa_J23115) | This study |
| *E. coli* W3110 MAGIC v.2 | *E. coli* W3110 P_tac_­*pglB* | This study |
| pUT-mini-Tn*5*Km2 | *ori* R6K, mob RP4, miniTn*5km-*based delivery with delivery plasmid with Amp^r^ | (33) |
| pGAB2 | *F. tularensis* O-antigen biosynthesis locus cloned in pLAFR-1 | (10) |
| pB4 | *S. pneumoniae* serotype 4 capsule locus (*wciI-fnlC*) pBBR1MCS-3 (Tc^R^ | (14) |
| pWA2 | *C. jejuni* Cme*A* with a *pelB* signal sequence and His6-tag. (Ap^R^) | (34) |
| pJAN25 | *ori* R6K, mob RP4, miniTn*5km-*based delivery with delivery plasmid with Amp^r^ coding for IPTG inducible *C. jejuni pglB* cloned in NotI site controlled by pTac promoter. | This study |
| pUA73 | *ori* R6K, mob RP4, miniTn*5Zeo-*based delivery with delivery plasmid with Amp^r^ coding for IPTG inducible *C. jejuni pglB* cloned in SfiI site carrying pTac promoter. Zeo^r^ cassette flanked by loxP sites | This study |
| pOST9 | pMB1 ori, Amp^r^ plasmid carrying IPTG inducible *C. jejuni pglB* | This study |
| pELLA1 | *ori* R6K, mob RP4, miniTn*5Zeo-*based delivery with delivery plasmid with Amp^r^ coding for IPTG inducible *C. jejuni pglB* cloned in NotI site controlled by constitutive promoter BBa_J23109 | This study |
| pELLA2 | *ori* R6K, mob RP4, miniTn*5Zeo-*based delivery with delivery plasmid with Amp^r^ coding for IPTG inducible *C. jejuni* p*glB* cloned in NotI site controlled by constitutive promoter BBa_J23114 | This study |
| pELLA3 | *ori* R6K, mob RP4, miniTn*5Zeo-*based delivery with delivery plasmid with Amp^r^ coding for IPTG inducible *C. jejuni* p*glB* cloned in NotI site controlled by constitutive promoter BBa_J23115 | This study |

**MAGIC construction**

The gene coding for *C. jejuni* NCTC11168 PglB was loaded into a Mini-Tn*5*Km2 transposon within a pUT backbone targeting the NotI site. However, in order to assemble a construct that could be induced, we first cloned *C. jejuni pglB* into pEXT20. This is a vector that enables IPTG inducible expression of ORFs that are inserted within the MCS ^14^. The gene coding for *C. jejuni* PglB was amplified by PCR from the plasmid pACYC*pgl* with the pTac promoter and LacIq repressor from plasmid pEXT20 as a template using Pfx Polymerase (Thermo Fisher Scientific UK) with (SEQ ID 15: 5′-TTTTGCGGCCGCTTCTACGTGTTCCGCTTCC-3′) as forward primer and (SEQ ID 16: 5′-TTTTGCGGCCGCATTGCGTTGCGCTCACTGC-3′) reverse primer using the following cycling conditions, 94 °C 2 min followed by 35 cycles of 94 °C for 30 sec, 56 °C for 30 sec and 68 °C for 4 min. The PCR product was then cloned in pJET2.0 plasmid (Thermo Scientific U.K.) according to the manufacturer's instructions and named pOST9. The plasmid was maintained in *E. coli* DH5α cells (Stratagene U.K.). The vector pOST9 was cut with the restriction enzyme NotI (New England Biolabs U.K. Ltd.) and ligated into the unique NotI site in pUTMini-Tn*5*Km2 resulting in plasmid pJAN25 and maintained in Transformax *E. coli* strain EC100D *pir*+ (Cambio U.K.). The plasmid was then transformed into *E. coli* 19851 *pir*+ or *E. coli* MFD for maintenance and for conjugation with host bacteria for delivery on *pglB*.

**Bacterial Conjugation**

To enable transfer of the *CjpglB* and *cmeA* from the transposon cargo into the chromosome of a recipient *E. coli* strain using the plasmids pJAN25 and pFEB11 respectively, the new loaded Mini-Tn*5*Km2 transposons were maintained in *E. coli* strain19851*pir*^+^. We switched to using *E. coli* MFD a diaminopimelic acid (DAP) auxotroph. Growth medium was supplemented with kanamycin 50 μg/ml and ampicillin 100 μg/ml for pJAN25 or pFEB11 whilst chloramphenicol 30 μg/ml and ampicillin 100 μg/ml were added to maintain pEFNOV19. Both donor and recipient bacteria were growth until late exponential phase. Bacterial cells were pelleted by centrifugation, washed 3 times with PBS and mixed together in a ratio of 1:3 recipient to donor and spotted on a dry LB agar plate with no antibiotics for 4-8 hrs. The cells were scraped and suspended in PBS and dilutions plated on LB agar with appropriate selection antibiotics to select for transconjugants. Individual colonies were picked up and screened for loss of the pUT backbone and for the presence of the transposon.

**Protein purification**

Protein purification was carried out using QIAExpressionist NiNTA purification kit according manufacturer’s instructions (Qiagen, Germany) and following the HIS purification under native conditions protocol. Purification was carried out from 10 ml of bacterial culture. If induced, bacterial cells from an o/n culture were used to inoculate 10 ml of Luria Bertani (LB) broth. Cells were incubated at 37 °C, 180 rpm until an OD_600_ of 0.4 was reached. At this point L-arabinose was added to a 0.2% v/v final concentration or IPTG at a 1mM final concentration. Samples were incubated at 37 °C for a further 16 h before the cell pellet was collected for protein purification. Cells were lysed in lysis buffer (50 mM NaH_2_PO4, 300 mM NaCl, 10 mM Imidazole, pH 8) following manufacturers protocol. Cells debris were removed by centrifugation at 12,000 xg, supernatant was incubated for an hour with NiNTA agarose at 4 °C then 4 times beads volume with wash buffer (50 mM NaH_2_PO4, 300 mM NaCl, 20 mM Imidazole, pH 8), then eluted with 50 mM NaH_2_PO4, 300 mM NaCl, 250 mM Imidazole, pH 8.

**Mass spectrometry**

In gel reduction, alkylation, and digestion with trypsin or chymotrypsin was performed on the gel sample prior to subsequent analysis by mass spectrometry. Cysteine residues were reduced with dithiothreitol and derivatized by treatment with iodoacetamide to form stable carbamidomethyl derivatives. Trypsin digestion was carried out overnight at room temperature after initial incubation at 37°C for 2 h. Sample digests, resuspended in 0.1% (v/v) formic acid, were analyzed by on-line nano-flow reverse-phase high-performance liquid chromatography with online electrospray-mass spectrometric analysis (nano-RP-HPLC-ES-MS) with MS/MS (MS^e^) using a Waters SYNAPT G2-S high-definition mass spectrometer, coupled to a Waters ACQUITY UPLC M-Class System (Waters UK, Elstree). Separations were achieved by means of a C18 trapping column (M-Class Symmetry C18 Trap, 100 Å, 5 μm, 180 μm × 20 mm, 2G) connected in-line with a 75 μm C18 reverse-phase analytical column (M-Class Peptide BEH C18, 130 Å, 1.7 μm, 75 μm × 150 mm) eluted over 90 min with a gradient of acetonitrile in 0.1% formic acid at a flow rate of 300 nL/min. Column temperatures were maintained at 50°C, and data were recorded in MS^e^ “Resolution” positive ion mode, with scan times set to 0.5 s in both the high-energy and low-energy modes of operation. The instrument was pre-calibrated using 10–100 fmol/μL of [Glu^1^]-fibrinopeptide B/5% (v/v) acetic acid (1:3, v/v) and calibrated during analysis by means of a lockmass system using [Glu^1^]-Fibrinopeptide B 785.8426^2+^ ion. The collision gas utilized was argon with collision energy ramp of 20–45 eV. Data acquisition was performed using MassLynx (Waters UK, Elstree) software and analyzed by means of MassLynx, BiopharmaLynx and ProteinLynx Global Server (PLGS) version 3.0.2 (Waters UK, Elstree).

**Cell free glycosylation**

Cell free glycosylation was conducted in S30 buffer with 0.1% n-dodecyl-β-d-maltopyranoside (DDM; Thermo Scientific) and 10 mM MnCl_2_ (Across Organics). A constant volume of lysed acceptor protein and glycan was mixed to varying volumes of lysed OSTs ranging from 100 μl to 500 μl for a total reaction volume of 1 ml**.** Cell free glycosylation was conducted at 30 °C and 110 rpm overnight. Afterwards, the samples were centrifuged at 12,000 xg for 10 minutes to remove reaction debris. The samples were then incubated for an hour and a half in Ni-NTA resin (Qiagen) at 4 °C to pull down glycosylated acceptor protein. After washing samples with His Wash buffer (50 mM NaH_2_PO4, 300 mM NaCl, 20 mM Imidazole, pH 8), the samples were eluted using 75 μl His Elution buffer (50 mM NaH_2_PO4, 300 mM NaCl, 250 mM Imidazole, pH 8).

**Supplementary method:**

**Assembly of constitutively expressed *C. jejuni* PglB**

Using *C. jejuni* 81116 genomic DNA as a template *pglB* was amplified using the forward primers

X4

5’-TTTTGCGGCCGCTTTACAGCTAGCTCAGTCCTAGGGACTGTGCTAGCAGGAGGAAAAAAATGTTGAAAAAAGAGTATTTAAAAA -3’ BBa_J23109

X10

5’-TTTTGCGGCCGCTTTATGGCTAGCTCAGTCCTAGGTACAATGCT

AGCAGGAGGAAAAAAATGTTGAAAAAAGAGTATTTAAAAA -3’

BBa_J23114

X15

5’-TTTTGCGGCCGCTTTATAGCTAGCTCAGCCCTTGGTACAATGCTAGCAGGAGGAAAAAAATGTTGAAAAAAGAGTATTTAAAAA -3’ BBa_J23115

X72

5’-TTTTGCGGCCGC TTGACAGCTAGCTCAGTCCTAGGTATTGTGCTAGCAGGAGGAAAAAAATGTTGAAAAAAGAGTATTTAAAAA-3’ BBa_J23104

REV *CjpglB*: 5’-TTTTGCGGCCGCTTAAATTTTAAGTTTAAAAACTTTAGC-3'

94°C/15 s, [94 °C/30 s, 50 °C/30s, 68 °C/2min]* 68°C/2min

*35 cycles

PCR products were digested with NotI HF (New England Biolabs) and cloned into NotI digested Cloned into pUC57ZeoTn.

The plasmid was then digested with SfiI and ligated to pUTminiTn*5* to create the plasmid pELLA1 (pglBx4), pELLA2 (pglBx10), pELLA3 (pglBx15). Conjugation to the *E. coli* strain CROW was carried out to deliver the constitutive *pglB* copy.
